# Neurofilaments in blood and CSF for diagnosis and prediction of onset in Creutzfeldt-Jakob disease

**DOI:** 10.1038/srep38737

**Published:** 2016-12-08

**Authors:** Petra Steinacker, Kaj Blennow, Steffen Halbgebauer, Song Shi, Viktoria Ruf, Patrick Oeckl, Armin Giese, Jens Kuhle, Dana Slivarichova, Henrik Zetterberg, Markus Otto

**Affiliations:** 1 Department of Neurology, Ulm University, Ulm, Germany; 2 Inst. of Neuroscience and Physiology, Dept. of Psychiatry and Neurochemistry, The Sahlgrenska Academy at University of Gothenburg, Mölndal, Sweden; 3 Clinical Neurochemistry Laboratory, Sahlgrenska University Hospital, Mölndal, Sweden; 4 Center for Neuropathology and Prion Research, Ludwig-Maximilians University, Munich, Germany; 5 Neurology, Departments of Medicine, Biomedicine and Clinical research, University Hospital Basel, Basel, Switzerland; 6 Department of Prion Diseases, Slovak Medical University, Bratislava, Slovakia; 7 Department of Molecular Neuroscience, UCL Institute of Neurology, Queen Square, London, UK

## Abstract

While cerebrospinal fluid (CSF) biomarkers for Creutzfeldt-Jakob disease (CJD) are established and partly included in the diagnostic criteria, no blood biomarkers are available. Here, we assessed the utility of serum neurofilament light chain (NF-L) and tau protein in comparison to CSF markers (NF-L and phosphorylated NF heavy chain (pNF-H), tau, S100B, 14-3-3) and prion conversion assay (real-time quaking induced conversion (RT-QuIC)) for sporadic and genetic CJD. Importantly, a Gerstmann-Sträussler-Scheinker mutation carrier in the asymptomatic phase and at disease onset was included as well. Both NF-L and tau were markedly increased in CJD serum, reaching similar or even better performance as in CSF (sensitivity and specificity for serum NF-L 100% and 85.5%, and for serum tau 84.6% and 96.2%, respectively). Serum S100B showed high sensitivity as well (84.2%), but lower specificity (63%). CSF neurofilaments were increased before symptom onset, while prion seeding assay was negative. Just before a clinical diagnosis could be made, all CSF markers and NF-L in the serum were increased and CSF prion conversion assay was positive. The data suggest that neurofilaments are sensitive and specific blood markers for the diagnosis of genetic and sporadic CJD and might represent promising tools to predict disease onset.

Prion diseases are transmissible and fatal neurodegenerative diseases neuropathologically characterized by the presence of misfolded prion proteins, which are exceptionally resistant to routine decontamination procedures. The availability of an easy, early, and reliable test in prion disorders is therefore a relevant issue both for public health reasons and for establishing diagnosis, but also for differential diagnosis with other treatable conditions.

The diagnosis of sporadic and genetic CJD can be supported by fluid biomarkers, including 14-3-3 proteins, tau proteins, neurofilaments and S100B in cerebrospinal fluid (CSF)^1^,^2^,^3^,^4^,^5^,^6^. Potential blood markers comprise acute-phase proteins^7^ and S100B^8^. Among the mentioned candidate markers, only 14-3-3 in the CSF was included into the diagnostic criteria by the WHO.

While for a long time the detection of misfolded prion protein in body fluids failed, some approaches have eventually been successful^9^ and high diagnostic accuracy was reported for the recently developed real-time quaking induced conversion (RT-QuIC) prion conversion assay which uses minute amounts of the β-structure-rich insoluble conformer of the prion protein (PrP^Sc^) in CSF as seeds^10^,^11^ while other studies report lower diagnostic performance^12^. Further, it is unclear when the misfolded prion protein appears or reaches the detectable limit in CSF of CJD patients. However, for disease onset, especially in genetic cases, blood markers are needed with the perspective to have an objective read-out for therapeutic trials. Presently, no validated blood biomarker for the differential diagnosis and prediction of onset exists.

In the present study we therefore addressed the questions whether we can measure these biomarkers with newly established assays in blood, of which diagnostic value they are, and how early in the disease process these markers appear.

## Results

A summary of the marker concentrations, cut-off levels, and diagnostic performance in terms of sensitivity and specificity as well as the positive and negative predictive values for the discrimination between CJD and controls is given in Table 1.

### Surrogate marker concentrations in serum

**Figure 1:**
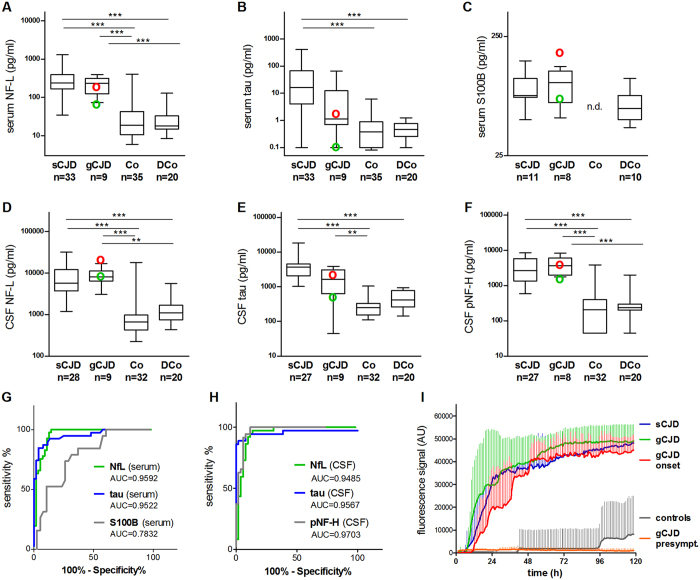
Marker concentrations, ROC diagrams, and RT-QuIC results for CJD and controls. Boxplots show the serum levels of NF-L (**A**), tau (**B**), and S100B (**C**), as well as the CSF levels of NF-L (**D**), tau (**E**), and pNF-H (**F**). Displayed are the results for CJD patients (indicated separately for sCJD and gCJD) and from demented (DCo) and non-demented controls (Co). The concentrations of analytes determined in serum and CSF from a gCJD patient at presymptomatic and early symptomatic disease stage are indicated by green and red circles, respectively. Boxes give median values with interquartile ranges, whiskers indicate the concentration range. Three and two asterisks indicate statistically significant differences of p < 0.0001 and p < 0.01, respectively. The diagnostic performance of markers is illustrated by ROC curves for serum (**G**) and CSF (**H**). Except for S100B, of which levels were determined in DCo only, ROC analyses were conducted considering all CJD measures (i.e. sCJD and gCJD) versus all controls (i.e. DCo and Co). The results of RT-QuIC are shown in (**I**). Displayed are the mean fluorescence signals with SD detected in the CSF of sCJD patients (n = 6), gCJD patients (n = 4), and controls (n = 6). Separately shown are the results for 3 replicate measurements of the gCJD mutation carrier in the asymptomatic disease stage (orange) and at disease onset (red).

Boxplots of marker levels in serum of the diagnostic groups are shown in Fig. 1A–C. NF-L serum concentrations were higher in both sCJD and gCJD compared to DCo or Co. At 44.7 pg/ml cut-off 100% sensitivity and 85.5% specificity was reached for discrimination between CJD and controls.

Serum tau was significantly different in sCJD, but not gCJD, and controls. At 2.2 pg/ml cut-off, CJD was recognized with 84.6% sensitivity and 96.2% specificity.

The concentrations determined for S100B in serum samples differed significantly between all CJD and DCo (p = 0.0006), but post hoc test after Kruskal-Wallis test (p = 0.0465) revealed no significant differences between sCJD, gCJD, and DCo (Fig. 1C).

At the presymptomatic stage serum tau and S100B were similar to control levels, whereas NF-L tended to be elevated, and at symptom onset serum markers were clearly increased (green and red circles respectively in Fig. 1A–C).

ROC analysis of serum marker concentrations of all CJD versus all controls yielded comparable estimates for the AUC for NF-L and tau (Fig. 1G).

### Surrogate marker concentrations in CSF

Boxplots of the marker levels in CSF samples of the diagnostic groups are shown in Fig. 1D–F.

The levels of NF-L and pNF-H in CSF samples were strongly increased in CJD compared to both control groups. CSF tau concentrations were significantly increased in CJD as well, however, due to low levels in an E200K and the insert 4 × 24 carrier, the difference between gCJD and DCo was not significant.

At the presymptomatic disease stage, CSF tau was normal and NF-L and especially pNF-H were already elevated above the 75% quartile of controls. At disease onset all CSF markers were increased. See green and red circles in Fig. 1D–F, respectively.

In the ROC analyses pNF-H, tau, and NF-L showed similar performance (Fig. 1H).

### Prion seeding activity in CSF

A summary of RT-QuIC results is shown in Fig. 1I. In 10/14 CJD CSF samples the RT-QuIC assay was positive (71.4% sensitivity). False negative results were obtained for one sample from a patient with M/M codon 129 polymorphism and an NF-L serum just above the cut-off level, and for 3 gCJD samples from patients with E200 K and E196 K mutations. These 3 patients were positive for CSF 14-3-3 and above the cut-off for all other markers, with the exception that the E200 K mutation carrier had a tau CSF level clearly below the diagnostic threshold. CSF samples from 6 non demented control cases failed in the RT-QuIC assay (100% specificity). After a prolonged amplification time, in 2 out of 6 control CSF samples a signal emerged after 48 h and 96 h, respectively, resulting in a signal less than 10% of the signal obtained with CJD samples after 5 days of measurements. In the CSF from the presymptomatic gCJD patient no seeding activity could be detected. At the onset of first symptoms the signal was positive with minimally delayed starting point of about 3 h and a maximum similar to that determined in the other positive CJD samples.

## Discussion

Today, improvement of CJD diagnosis is attempted in two ways: (1) There is a search for surrogate markers in the serum of CJD patients, and (2) Methods are developed to amplify the minute amounts of abnormal PrP present in body fluids for a specific diagnosis.

In the present study we show that the known biomarkers NF-L and tau measured in serum samples parallel the increase found in CSF, yielding similar statistical significance for differentiation. NF-L, which also is elevated in motor neuron disease^13^,^14^,^15^, is found at even higher levels in CJD patients. pNF-H which was found to be increased also in amyotrophic laterla sclerosis (ALS) plasma by in house assays^16^,^17^, yielded excellent diagnostic performance in CSF in our cohort. Additionally, the analysis of the serum from a presymptomatic gCJD patient provides first evidence for pNF-H to be elevated already before symptoms appear and clinical diagnosis can be made.

If serum NF-L qualifies as marker for broad differential diagnoses has to be examined in future studies including especially patients with rapidly progressive neurodegenerative dementia. As recent data point to a generally strong correlation between CSF and blood levels of NF-L and proteopathic lesions^18^, high diagnostic power for the discrimination of CJD and at least neurodegenerative diseases is likely to be expected.

Serum tau can now be detected more sensitively and is shown for the first time to be increased not only in sCJD^19^ but also in gCJD. Differences between gCJD and controls, however, were less marked compared to NF-L and pNF-H.

The time point at which the biomarkers increase in CSF and also in serum is a mostly open question. It can be hypothesized that the analyzed markers are increased early in CJD as they indicate neuroaxonal degeneration. We found the presymptomatic stage of one CJD mutation carrier characterized by normal serum and CSF tau and serum S100B. Neurofilaments, especially pNF-H in the CSF, already show a trend for increased levels and might therefore represent candidate markers for the onset of the disease as it was shown for asymptomatic ALS gene carrier^15^. At symptom onset of the gCJD patient, all markers are clearly increased in serum and CSF to the range of the CJD cohort.

Similar results were obtained with the specific approach, the RT-QuIC, which has been established for CJD diagnostics from CSF^12^,^20^,^21^ and urine^22^, however, failed to be successful in serum until now. Seeding activity could not be detected in the CSF from the presymptomatic phase, but in parallel to the appearance of first CJD symptoms.

Taken together, we show that serum NF-L and serum tau can be used in the diagnosis of both sCJD and gCJD and provide preliminary evidence that neurofilament levels tend to be increased presymptomatically.

## Methods

### Patients

This study was conducted according to the principles expressed in the Declaration of Helsinki. Collection and analysis of samples were approved by the Ethics Committees of the Medical Faculties of the University Göttingen (approval number 100305) and Ulm (approval number 20/10). All patients or their next relatives in case of severe dementia gave written informed consent to their participation in the study.

In total, our retrospective study included 103 patients that were seen in the general outpatient clinic and the outpatient memory clinic of the Department of Neurology in Ulm and the surveillance unit for transmissible spongiform encephalopathies in the Department of Neurology in Göttingen.

All methods were performed in accordance with the relevant guidelines and regulations.

CSF was obtained by lumbar puncture, centrifuged, aliquoted and stored within 2–48 h at −80 °C until analysis. Serum was processed likewise and stored within 2 h at −80 °C.

Serum and CSF samples of 43 CJD patients were analyzed, of which 4 had clinically diagnosed “probable CJD” (neuropathology missing) and 39 were neuropathologically verified, including 33 sporadic CJD (sCJD) and 9 genetic CJD (gCJD: E200 K (n = 3), V210I (n = 2), E196 K, insert 5 × 24 (n = 3), and insert 4 × 24). Additionally, samples from a 50 year old GSS patient (P102L) at the presymptomatic disease stage and two years later at onset of symptoms before clinical diagnosis were included. Control groups comprised 40 patients without signs of dementia (Co), and 20 demented patients (DCo) (Alzheimer’s disease n = 12, mild cognitive impairment n = 4, frontotemporal dementia n = 3, normal pressure hydrocephalus n = 1).

For demographic characteristics of the diagnostic groups see Table 1.

### Laboratory markers

Serum NF-L and serum tau protein were measured with ultrasensitive Single molecule array (Simoa) assays^23^,^24^,^25^.

Samples were analyzed for S100B with electrochemiluminescence immunoassay (Elecsys S100B, Roche, Penzberg, Germany)^8^, and with ELISAs for NF-L (IBL, Hamburg, Germany), pNF-H (Biovendor, Heidelberg, Germany), and Tau (Fujirebio, Hanover, Germany) according to manufacturer’s specifications. Mean inter-assay CV for the ELISAs was <20%^14^.

Real-time quaking-induced conversion (RT-QuIC) was carried out as described elsewhere^21^.

### Statistics

Statistical analyses were performed using graphpad prism 5 software. Standard measures of diagnostic test validity such as sensitivity, specificity, and predictive values accompanied by their 95% confidence intervals (CI) were calculated for varying biomarker cut-off levels. The optimal cut-off level for dichotomizing values was selected as the situation maximizing the Youden index^26^. The receiver operating characteristics (ROC) curve is used for a graphical visualization of the impact of the variation in the cut-off values. Two-tailed unpaired Mann-Whitney t-test and Kruskal-Wallis test at a significance level of 5% were used to determine statistical differences between two groups or more groups, respectively. Dunn’s post hoc comparison was applied following Kruskal-Wallis test in case of significant differences.

## Additional Information

**How to cite this article**: Steinacker, P. *et al*. Neurofilaments in blood and CSF for diagnosis and prediction of onset in Creutzfeldt-Jakob disease. *Sci. Rep.* **6**, 38737; doi: 10.1038/srep38737 (2016).

**Publisher's note:** Springer Nature remains neutral with regard to jurisdictional claims in published maps and institutional affiliations.

## Figures and Tables

**Table 1:** Demographic characteristics of the patient cohort, marker concentrations in serum and CSF, and diagnostic performance.

	n	age (years)^a^	gender (f/m)	serum NF-L (pg/ml)^b^	serum tau (pg/ml)^b^	serum S100 (pg/ml)^b^	CSF NF-L (pg/ml)^b^	CSF tau (pg/ml)^b^	CSF pNF-H (pg/ml)^b^	14-3-3 pos./neg.	RT-QuIC pos./neg
Co	40	64 (59–69)	24/16	49.2 ± 81.5	0.8 ± 1.2	68.6 ± 55.4	1632 ± 3328	293 ± 213	398 ± 759	*n.d.*	0/6 n = 6
DCo	20	64(62–67)	10/10	25.7 ± 25.7	0.6 ± 0.3	66.6 ± 20.5	1502 ± 1234	502 ± 266	331 ± 437	*n.d*.	*n.d.*
CJD	43	63(58–71)	23/22	296.9 ± 253.0	54.2 ± 90.7	95.8 ± 31.6	8908 ± 7056	2080 ± 530	3602 ± 2393		10/4 n = 14
sporadic CJD^c^	33	63 (58–80)	20/13	317.2 ± 282.3	67 ± 100	97.4 ± 31.4	8087 ± 6469	2152 ± 320	3255 ± 2254	28/5	6/1^d^ n = 7
	24 M/M	63(47–80)	15/9	274.0 ± 182.0	58.4 ± 95.1	101.2 ± 23.0	8242 ± 6817	4696 ± 3893	3252 ± 2048	21/3	4/1 n = 5
	3 M/V^e^	66(58–79)	3/0	88.8; 1038.7; 1308.4	2.7; 6.0; 275.7	n.d.	5056; 17985	2312; 3693	594; 7740	1/2	*n.d.*
	1 V/V^f^	64	m	313.6	4.1	50	32190	4245	8514	pos.	pos.
genetic CJD	9	63 (54–73)	3/8	222.8 ± 111.4	11.0 ± 21.5	99.5 ± 31.34	8784 ± 4239	1939 ± 1426	4115 ± 2342	7/3	4/3
	E196K	72	m	133.4	5.0	96	17155	3360	8310	pos.	neg.
	E200K	75	m	234.3	65.9	131	8400	340	6654	pos.	neg.
	E200K	45	m	72.9	0.1	109	13920	2478	4490	pos.	pos.
	E200K	72	m	279.8	1.2	52	5957	1657	2460	pos.	neg.
	insert (5 × 24)	63	m	146.1	19.8	*n.d.*	8265	<45	*n.d.*	neg.	*n.d*.
	insert (5 × 24)	73	m	393.8	4.6	140	7043	*n.d.*	4185	neg.	pos.
	insert (4 × 24)	57	f	115.2	1.1	120	7209	3902	1866	pos.	pos.
	V210I	77	f	345.3	0.5	83	3056	907	1761	pos.	pos.
	V210I	54	m	284.3	0.9	65	8050	2824	3192	pos.	*n.d*.
	P102L presympt.	50	f	63.6	*n.d.*	74	8146	459	1500	*n.d*.	neg.
	P102L onset	52		189.1	*n.d.*	181	20690	1969	4089	neg.	pos.
cut-off^g^				>44.70	>2.195	>64.00	>2156	>987.5	>562.5		
sensitivity^g^				100% (69.5–94.1)	84.6% (69.5–94.1)	84.2% (60.4–96.6)	97.3% (85.8–99.9)	88.9% (73.9–96.9)	100.0% (90–100)	*n.a.*	71.4% (41.9–91.6)
specificity^g^				85.5% (86.8–99.5)	96.2% (73.3–93.5)	63.2% (46–78.2)	86.5% (74.2–94.4)	98.1% (89.7–100)	88.5% (76.6–95.7)	*n.a.*	100% (55.1–100)
PPV^g^				0.837 (0.698–0.922)	0.920 (0.764–0.926)	0.790 (0.539–0.930)	0.818 (0.668–0.913)	0.970 (0.825–0.998)	0.854 (0.701–0.939)	*n.a.*	1 (0.656–1)
NPV^g^				0.979 (0.875–0.999)	0.853 (0.733–0.927)	0.6 (0.274–0.863)	0.978 (0.868–0.999)	0.927 (0.816–0.976)	1 (0.904–1)	*n.a.*	0.6 (0.274–0.863)

Co, non-demented control; DCo, demented control; f, female; m, male; pos., positive; neg., negative; n.d., not determined; n.a., not applicable; PPV, positive predictive value; NPV. negative predictive value. ^a^The age is given as median with interquartile range. ^b^The markers are given as mean with standard deviation. ^c^Codon 129 polymorphism was determined for 28 sporadic CJD cases. ^d^For 5 out of 7 CJD analyzed for CSF prion seeding activity the codon 129 polymorphism was known. ^e^For the patients with M/V individual marker concentrations are given; CSF parameters missing for one patient. ^f^This patient also had an A117A polymorphism. ^g^Calculated for discrimination between all CJD versus all control measures; given with 95% confidence intervals in brackets.
